# Functional Analysis With a Barcoder Yeast Gene Overexpression System

**DOI:** 10.1534/g3.112.003400

**Published:** 2012-10-01

**Authors:** Alison C. Douglas, Andrew M. Smith, Sara Sharifpoor, Zhun Yan, Tanja Durbic, Lawrence E. Heisler, Anna Y. Lee, Owen Ryan, Hendrikje Göttert, Anu Surendra, Dewald van Dyk, Guri Giaever, Charles Boone, Corey Nislow, Brenda J. Andrews

**Affiliations:** * Department of Molecular Genetics, University of Toronto, Ontario M5S 1A8, Canada; † Banting and Best Department of Medical Research, University of Toronto, Ontario M5G 1L6, Canada; ‡ Donnelly Centre, University of Toronto, Ontario M5S 3E1, Canada, and; § Department of Pharmaceutical Sciences, University of Toronto, Ontario M5S 3M2, Canada

**Keywords:** barFLEX array, gene overexpression, barcoders, synthetic dosage lethality

## Abstract

Systematic analysis of gene overexpression phenotypes provides an insight into gene function, enzyme targets, and biological pathways. Here, we describe a novel functional genomics platform that enables a highly parallel and systematic assessment of overexpression phenotypes in pooled cultures. First, we constructed a genome-level collection of ~5100 yeast barcoder strains, each of which carries a unique barcode, enabling pooled fitness assays with a barcode microarray or sequencing readout. Second, we constructed a yeast open reading frame (ORF) galactose-induced overexpression array by generating a genome-wide set of yeast transformants, each of which carries an individual plasmid-born and sequence-verified ORF derived from the *Saccharomyces cerevisiae* full-length EXpression-ready (FLEX) collection. We combined these collections genetically using synthetic genetic array methodology, generating ~5100 strains, each of which is barcoded and overexpresses a specific ORF, a set we termed “barFLEX.” Additional synthetic genetic array allows the barFLEX collection to be moved into different genetic backgrounds. As a proof-of-principle, we describe the properties of the barFLEX overexpression collection and its application in synthetic dosage lethality studies under different environmental conditions.

Deletion or overexpression of most yeast genes has little effect on cell fitness (Giaever *et al.* 2002; Sopko *et al.* 2006); however, a phenotype associated with the perturbation of a particular query gene often can be revealed in specific genetic backgrounds, such as those defective for functionally related genes (Kroll *et al.* 1996; Measday *et al.* 2005; Sopko *et al.* 2006). To systematically address genetic buffering, we developed methods for global mapping of genetic interaction networks. In particular, the synthetic genetic array (SGA) method automates the analysis of yeast genetic interactions, enabling the systematic exploration of gene function through genetic network analysis (Dixon *et al.* 2008; Tong *et al.* 2001, 2004). SGA has been used extensively to map digenic interactions among deletion alleles of the ~5000 nonessential yeast genes (Costanzo *et al.* 2010; Tong *et al.* 2004). Double mutants with a more severe fitness defect than expected (based on a model for the combined fitness of the individual single mutants) represent a negative genetic interaction, with synthetic lethality as the most extreme case. SGA methodology also has been adapted to map synthetic dosage lethal (SDL) interactions quantitatively (Kaluarachchi *et al.* 2012; Sharifpoor *et al.* 2012), which occur when gene overexpression is of little consequence in a wild-type (WT) cell but causes a severe phenotype (*e.g.*, lethality) in a specific mutant background [(Kroll *et al.* 1996) supporting information, Figure S1].

Genome-wide SDL screening of yeast kinase mutants has identified new targets and regulators of kinases (Sharifpoor *et al.* 2012; Sopko *et al.* 2006). The kinome SDL screens revealed that most kinase deletion mutants are resistant to gene overexpression but that biologically meaningful genetic interactions could be discovered when screens were performed under conditions in which the kinase is active. For example, SDL screening of the high osmolarity responsive kinase, Hog1, revealed no interactions in standard growth conditions but identified 74 SDL interactions when assessed in the presence of 0.2 M sodium chloride (Sharifpoor *et al.* 2012). This finding highlights the need for developing efficient methods for parallel analysis of gene overexpression phenotypes in diverse conditions.

In budding yeast, a number of functional genomic resources are available that enable systematic analysis of SDL phenotypes through conditional induction of gene overexpression. These collections include: (1) a set of yeast strains each carrying a unique galactose-inducible N-terminal glutatione *S*-transferase (GST)-tagged open reading frame [ORF (Sopko *et al.* 2006; Zhu *et al.* 2000)]; (2) the movable ORF (mORF) strain collection, each carrying a unique galactose-inducible C-terminal HA- and protein A-tagged ORF (Gelperin *et al.* 2005); and (3) a set of yeast strains each carrying a unique galactose-inducible Flag epitope-tagged ORF [(Breitkreutz *et al.* 2010; Ho *et al.* 2002) Table 1]. Although each of these collections represent powerful resources for SDL analysis, they all contain tags that may compromise gene function, none of them carry fully sequenced-verified ORFs, and none of the strains are barcoded for highly parallel analysis in pooled cultures.

**Table 1:** Summary of overexpression resources available for *Saccharomyces cerevisiae*

Collection	Copy No.	Promoter	Sequence Verified?	Tag	No. ORFs	Yeast Marker	Array Available as
GST	High	GAL1/10 inducible	Some	N-GST	5280	*URA3*, *leu2d*	Yeast and *E. coli*
Sopko *et al.* 2006	2 μ						
Zhu *et al.* 2000							
mORF	High	GAL1/10 inducible	Yes (both ends of ORFs)	C-His_6_-HA-ProteinA	5854	*URA3*	Yeast and *E. coli*
Gelperin *et al.* 2005	2 μ					Gateway	
FLEX	Low	GAL1/10 inducible	Yes (fully verified)	None	5532	*URA3* Gateway Barcoded	Yeast and *E. coli*
Hu *et al.* 2007	CEN						
This study							
Tyers	Low	GAL1/10 inducible	No	C-Flag	1558	*LEU2*	*E. coli*
Ho *et al.* 2002	CEN						

ORF, open reading frame; GST, glutatione *S*-transferase; mORF, movable ORF; FLEX, full-length EXpression-ready; CEN, centromeric.

To circumvent the aforementioned limitations with existing yeast arrays, we combined the sequence-verified Full-Length EXpression-ready (FLEX) plasmid library of galactose-inducible untagged ORFs (Hu *et al.* 2007) with a set of Barcoder yeast strains, which are compatible with parallel competitive growth analysis. The strains in our resultant barcoded FLEX (barFLEX) array carry a unique oligonucleotide identifier integrated at a neutral locus (*ho*) and a specific *GAL1-ORF* plasmid. In a proof-of-principle analysis, we describe a robust protocol for using the barFLEX collection to explore fitness defects caused by gene overexpression in pooled cultures under different genetic or environmental perturbations.

## Materials and Methods

### Yeast strains used in this study

For a summary of the yeast strains used, see Table S1.

### Assembly of the yeast FLEX array

The plasmids from the FLEX collection (Hu *et al.* 2007) were mini-prepped in 96-well format from bacterial stocks using the Nucleospin Multi-96 Plus Plasmid Kit (Macherey-Nagel; cat. no. 740625.24). For quality control, we used capillary sequencing to assess the identity of the plasmid stocks. We found 3 of 76 plasmids were incorrect, giving an inherent error rate of 4% in the stocks we prepared from the FLEX collection strains (http://www.hip.harvard.edu/). We also tested 55 random strains from the SGA-FLEX array by polymerase chain reaction (PCR) amplification and restriction digestion of the ORFs to ensure that each ORF was represented on the correct location on the array. Almost all amplified ORFs (96%) appeared correct by one or both assays (data available on request).

Samples (2 μL) of each plasmid were transformed into Y6897 using standard lithium acetate and polyethylene glycol protocol (Gietz and Schiestl 2007). The transformants were selected on synthetic minimal media supplemented with glucose but lacking uracil (SD-URA) and then patched onto rectangular agar plates (OmniTray; Nunc International). The strain background for this collection was chosen such that the collection can be mated to the Barcoders and carried through the SGA protocol. The resulting diploids were sporulated and selected to yield *MAT*α haploid strains. The media used throughout SGA were prepared as described previously (Tong and Boone 2006). The transformants were patched in 96-well format with empty spaces left at the edges to allow for adding negative control “border” strains (Baryshnikova *et al.* 2010).

### Expansion of the Barcoder collection

To expand the original set of 1141 Barcoders (Yan *et al.* 2008), 3974 new Barcoder strains were constructed to accommodate all 5336 ORFs in the yeast SGA-FLEX array. PCR products carrying 2 unique barcodes flanking the *kanMX* resistance cassette were transformed into the BY4741 strain background as described previously (Yan *et al.* 2008). The PCR products were derived from 2 sources. A total of 3661 PCR products had been amplified from the nonessential yeast deletion collection using a common priming sequence (Dowell *et al.* 2010). To supplement these cassettes, 313 new pairs of primers were used to provide 313 additional amplicons for transformation and Barcoder generation. All 3974 PCR products were reamplified using the forward and reverse primers described previously (Yan *et al.* 2008) to direct all of the products to the *ho* locus in BY4741. Specifically, we used the following UPTAG and DOWNTAG primer pairs to amplify the kanamycin resistance cassette with 20-mer barcodes, whereby U2 and D1 are homologous to the *kanMX* cassette, and U1 and D2 are homologous to the *ho* locus (Figure S2):

Uptag: U1-GATGTCCACGAGGTCTCT; U2-CGTACGCTGCAGGTCGAC;
Downtag: D1-CGGTGTCGGTCTCGTAG; D2-ATCGATGAATTCGAGCTCG

PCRs were performed using Platinum PCR Supermix High Fidelity (Invitrogen). The transformants were selected by replica-plating onto standard yeast peptone dextrose (YPD) + G418 (200 mg/L Geneticin; Invitrogen) and patched onto agar plates in a 96-colony format. The Barcoder array was designed to pair perfectly with the FLEX array except for a small number of negative controls that were eliminated through SGA.

### Validating the barcode sequences in the Barcoder collection

Genomic DNA from the yeast Barcoder strains was isolated from a pool of the Barcoder collection. UPtag and DNtag PCR products were amplified separately; purified, mixed in equal molar ratios; and sequenced on an Illumina GAIIx, as described previously (Smith *et al.* 2009, 2010). In brief, barcode sequences were compared against the dataset generated previously (Smith *et al.* 2009) to determine whether additional annotation of barcode sequence was required. During sequencing, instead of using the standard Illumina sequencing primer, we used the yeast common primers that were adjacent to the Illumina sequencing primer. We used one primer for the UPtags (U1) and one primer for the DNtags (D2), each at a 10 μM concentration (Figure S3).

### Assembly of the barcoded FLEX collection (barFLEX)

The FLEX collection was crossed to the new Barcoder collection by robotic replica pinning on a Virtek Colony Arrayer (Bio-Rad Laboratories) in 384 format. The array was mated on YPD omni-trays by pinning once from each collection to the same plate. SGA was performed as described previously (Tong and Boone 2006) with the following selection media: two diploid selections (SD-URA+G418), selection on sporulation media, three haploid selections (SD-LEU-URA-ARG-LYS+Canavanine+Thialysine+G418). Haploid strains with the mating type *MAT*α were selected by excluding leucine from the medium (only *MAT*α strains will grow due to activation of the α-specific promoter fusion, *STE3*pr-*LEU2*). At this step, *MAT***a** strains also could be selected by changing the media to SD-HIS-URA-ARG-LYS+Canavanine+Thialysine+G418 (only *MAT***a** strains grow due to activation of the **a**-specific promoter fusion, *STE2*pr-*S.p.his5*). The barFLEX collection was maintained in the *MAT*α state to allow for subsequent rounds of SGA with *MAT***a** query deletion strains.

### Pooled growth experiments

The pooled yeast cultures were created according to previously described methods (Pierce *et al.* 2007). Pools were allowed to recover from −80° freezer stocks in SD-URA-LEU liquid medium for 5 hr before the liquid growth experiment was performed. Cultures were then diluted to OD 0.06 in either SD-URA-LEU or SG-URA-LEU liquid and grown for 20 generations. The cultures were grown for five generations and then diluted into new media a total of three times. Cell aliquots were saved every five generations on a cold plate and then pelleted and frozen for genomic DNA extraction. After PCR amplification of the barcodes using a set of common primers, the PCR product was hybridized to the TAG4 microarray (Pierce *et al.* 2006). The signal intensity of each barcode was quantified to determine the fitness of each strain. Only one of the two barcodes for each strain was used for analysis based on which tag gave the best signal intensity and number of reads. The signal from the UPTAGs and DNTAGs was normalized separately using quantile normalization. The signal from the growth in glucose was compared with the growth in galactose, and the resulting Log_2_ ratios (treatment/control) were used for analysis. Therefore, strains with decreased abundance in the pool show negative ratios. Pooled experiments in the presence of methyl methanesulfonate (MMS; Sigma-Aldrich) were performed by including either 0.001% or 0.0001% MMS in the growth medium.

### Kinase deletion screens

Two deletion alleles (*ura3Δ* and *dun1Δ*) were created in the BY4741 background to cross to the barFLEX array. The deletion alleles were marked with *natMX* providing resistance to nourseothricin (NAT). The *ura3Δ*::*natMX* and *dun1Δ*::*natMX* deletion strains were crossed to the barFLEX array using robotic pinning and selection through SGA as follows: 2X diploid selection SD-URA+G418 +NAT, 1X sporulation media, 2X haploid selection SD-LEU-URA-ARG-LYS+Canavanine+Thialysine+G418+NAT. Colonies from the final haploid selection plates were pooled and frozen at −80° before testing.

### Yeast serial spot dilutions

Confirmations of genetic interactions were performed using a serial spot dilution assay. Yeast cultures were grown to saturation overnight and then diluted 10-fold, five times. Five microliters of the dilutions was plated on glucose and galactose using a Biotek Precision 2000 robot and grown at 30° for 2 and 3 d, respectively, before imaging.

### Liquid growth curve assays

SDL interactions identified in the *dun1Δ*::*natMX* screen were confirmed using high-resolution growth curves in a TECAN spectrophotometer. Cultures were grown in minimal glucose media to saturation then diluted with a 96-well pin tool in minimal galacose media and grown at 30°. Optical density measurements were recorded every 15 min, while shaking for 2 d. Cultures were grown in triplicate and the average G measurements were recorded. The average G measures the rate of growth as the slope of a growth curve of a particular strain during log phase. The raw fitness defect caused by overexpression was measured by normalizing average G values to the negative control [*dun1Δ*::*natMX* overexpressing *FLEX-HIS3* plasmid (St Onge *et al.* 2007)]. Strains with a fitness defect greater than 10% and twice the standard deviation were considered SDL with *dun1Δ*::*natMX*.

## Results and Discussion

### Construction of the yeast FLEX array (SGA-FLEX)

**Figure 1:**
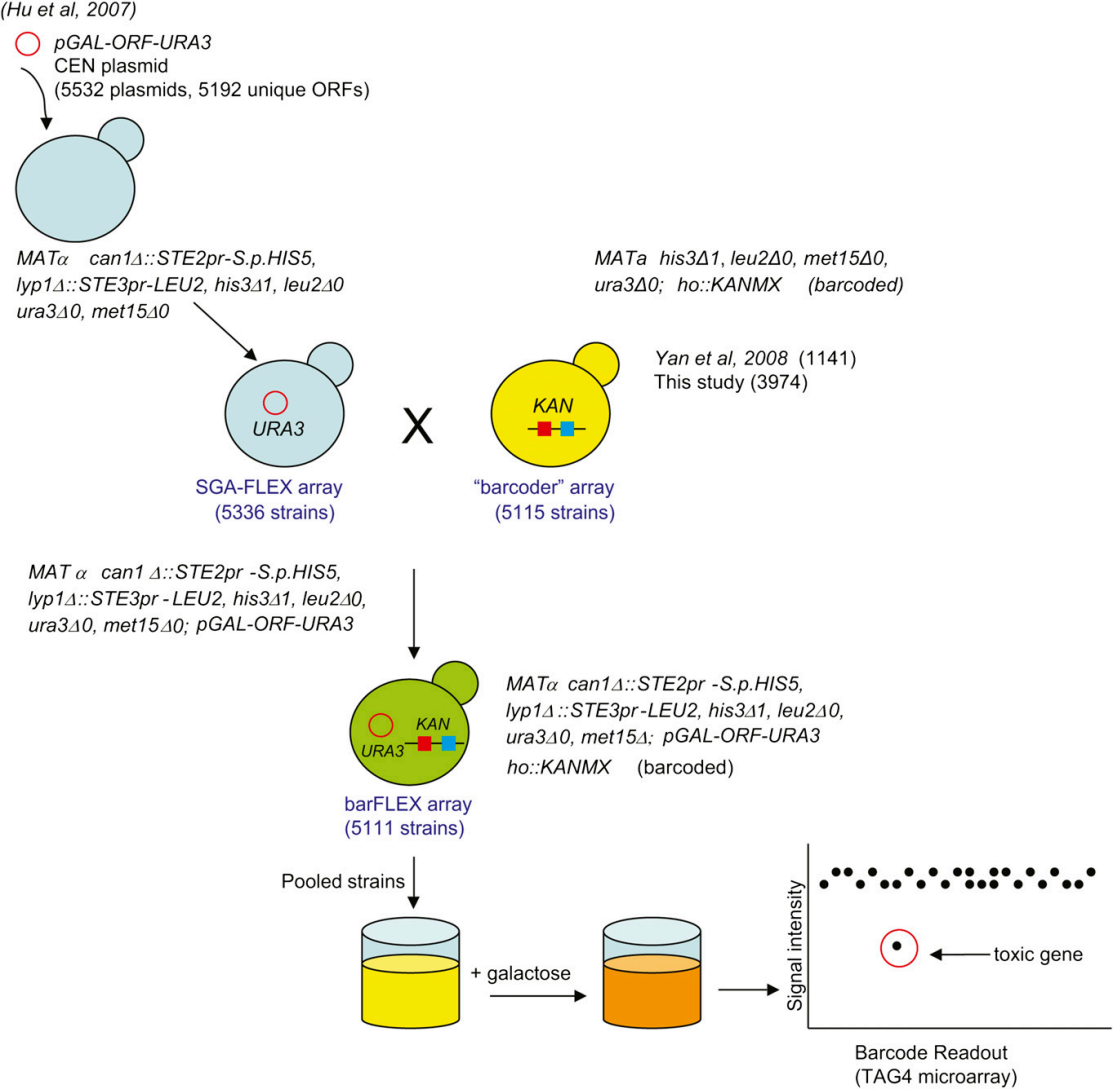
Construction of the barFLEX array. Step1: SGA using robotic pinning pairs each FLEXGene overexpression plasmid (pGAL-ORF) with a unique barcode-carrying strain. The FLEXGene plasmids are marked with a *URA3* selectable marker and the Barcoder strains are marked with a *kanMX* resistance marker. This selection assembles an inducible barcoded overexpression array after only one round of SGA.

To construct a yeast gene overexpression array in a genetic background that is compatible with SGA technology, we made use of the Yeast FLEX array, a unique collection of Gateway-compatible plasmids carrying sequence-verified yeast ORFs (Hu *et al.* 2007). We used a version of the FLEX collection in which each of 5192 unique ORFs (5532 plasmids in total) is expressed from the galactose-inducible *GAL1/10* promoter on the *URA3*-based CEN plasmid pBY011 (Hu *et al.* 2007). We purified plasmid DNA from the bacterial transformants harboring the FLEX plasmids and used them to transform yeast strain Y6897 [*MAT*α *his3Δ1 leu2Δ0 ura3Δ0 met15Δ0 can1Δ*::*STE2pr-Sp_his5 lyp1Δ*::*STE3pr-LEU2* (Tong and Boone 2006)], which is compatible with both SGA technology and the Barcoders collection (Figure 1) (Yan *et al.* 2008). The transformed strains were arrayed in 96-well format plates (n = 72) and then consolidated into 18 different 384-well plates using robotic pinning to create the SGA-FLEX array (Table S2).

### Expanding the Barcoder collection

The use of pooled cultures of differentially barcoded mutant strains for genetic screens has a wide range of applications and offers particular advantages for chemical genetics, where small amounts of expensive and scarce chemicals can be screened for effects on the fitness of mutant strains (Hillenmeyer *et al.* 2008; Ho *et al.* 2009; Pierce *et al.* 2007; St Onge *et al.* 2007). Parallel analysis of pooled cultures also enables rapid analysis of cellular fitness in multiple environmental conditions. Previously, we expanded the tool-kit of barcoded reagents available in yeast to include a genomic collection of 1141 arrayed strains (Barcoders) with unique integrated barcodes at the same neutral locus, *ho* (Yan *et al.* 2008). We expanded the number of strains in the Barcoder collection to permit genome-wide assays and to allow full coverage of the SGA-FLEX collection. Our protocol for constructing Barcoder strains involved PCR-amplification of barcodes from the original YKO (yeast knockout) array, which contains two barcodes linked to each mutant locus (Giaever *et al.* 2002; Yan *et al.* 2008). To generate the initial collection of Barcoders, we selected the 2282 unassigned barcodes that were present on the commercially available TAG4 microarray (Pierce *et al.* 2006) and incorporated them in pairs into 1141 tagged Barcoder strains. To augment the collection, we selected an additional 7948 barcodes present on the TAG4 microarray. Most of these additional barcodes have been used successfully in studies with the yeast deletion mutant collection (Pierce *et al.* 2006). We used primers that recognize the flanking sequence of the barcoded cassettes as well as homology to the *ho* locus to produce PCR products for directed gene replacement into the *ho* locus in the *MAT***a** WT strain BY4741. The new Barcoder strains (3974) were rearrayed together with the 1141 original strains to create a final array of 5115 Barcoder strains in a format compatible with the SGA-FLEX array (Barcoder v2; Table S3).

### Generating the barFLEX collection

We next used the Barcoder v2 collection to efficiently add barcode identifiers to strains in the SGA-FLEX array (Figure 1). In brief, we used the SGA method to select for haploid strains carrying one overexpression plasmid and an integrated barcode as described previously (Yan *et al.* 2008). The Barcoder concept is based on the principle that a barcode and any marked genetic element of interest (in this case an overexpression plasmid) do not need to be genetically linked but must only appear in the same strain in order to track that strain’s behavior (*e.g.*, fitness) in a pool. The Barcoder v2 collection can be thus used to “barcode” any SGA-compatible collection of yeast strains. We validated our barFLEX collection by sequencing 54 randomly selected ORFs and 25 uptags. In this test, 96% of barcodes and 96% of ORFs were in the correct strain and in the expected location on the strain array (Lists of ORFs and barcodes tested available on request; Table S4)

In summary, our barFLEX collection contains 5111 strains (Table S5). The collection can be manipulated to generate either *MAT***a** or *MAT*α backgrounds, depending on the applied final haploid selection. The barFLEX strains are compatible with the SGA screening platform to introduce marked alleles of any genes of interest for combinatorial overexpression genetics.

### Characterization of the barFLEX array

To assess the performance of our barFLEX collection in pooled competitive growth assays, we generated *MATα* barFLEX strains and then pooled them as described previously (Pierce *et al.* 2007). As an initial test of the quality of the barcodes, we grew the pool in either 2% glucose or 2% galactose for 20 generations. We isolated genomic DNA at the end of the growth period, amplified the barcodes, and then hybridized the amplicons to the TAG4 microarray (Pierce *et al.* 2007). In total, ~80% of the Barcoder strains had signal intensities for both barcodes that were three times greater than the background hybridization intensity levels, consistent with our previously published experiments using the TAG4 array (Pierce *et al.* 2006; Yan *et al.* 2008). When we asked what fraction of Barcoder strains had at least 1 barcode that satisfied our detection criteria, this fraction increased to ~90% (Table S5 and Table S6). The undetected barcodes below background (Table S7) reflect those that contain errors in either their common priming sequences or barcodes (Smith *et al.* 2009).

Due to the ever-decreasing costs of next-generation sequencing, we previously compared barcode microarray data to “bar-seq” data and found them to be generally equivalent (Smith *et al.* 2010). We confirmed the sequences for all the barcodes within the Barcoder collection by sequence analysis as follows (sequence data can be accessed at http://chemogenomics.med.utoronto.ca/supplemental/barflex/). We assessed the presence of barcodes in the pool by next generation sequencing. The reads generated by the sequencing of DNA prepared from the pool in a single Illumina flow cell were counted and assigned to the barcodes expected to be in the pool. Global sequence alignments were generated for each of the unidentified barcodes to all unclassified reads. Reads were associated with barcodes for which alignment scores exceeded 80 (+5 match, −1 mm/indel; Table S8 and Table S9)

Of the 5106 upstream barcodes, 2518 (49%) were identified as a perfect match and another 1774 (35%) were matched by alignment. For the 5038 downstream barcodes the numbers of perfect matches and aligned reads were 2435 (48%) and 1796 (36%), respectively. Considering both barcodes for the 5106 individual barcoder strains, 1777 (35%) had direct matches for both barcodes and 3176 (62%) for at least one barcode. At least one barcode was identified by alignment in 1506 (22%) strains. For 807 strains (8%), neither barcode was clearly seen in the sequence data generated from this particular sequenced sample. Because these percentages are derived from a single sample, we expect that additional strains will be detectable with further algorithm development (*e.g.*, which accounts for PCR-induced variation as well as flow cell geography) and the supporting website will be updated to include these additional strains. We conclude that the barcodes of the barFLEX collection are suitable for analysis using either next generation sequencing or microarrays. We have generated a list of high scoring alignments of all unique reads in our sequence data (http://chemogenomics.med.utoronto.ca/supplemental/barflex/).

### Detecting overexpression toxicity using pooled cultures derived from barFLEX

**Figure 2:**
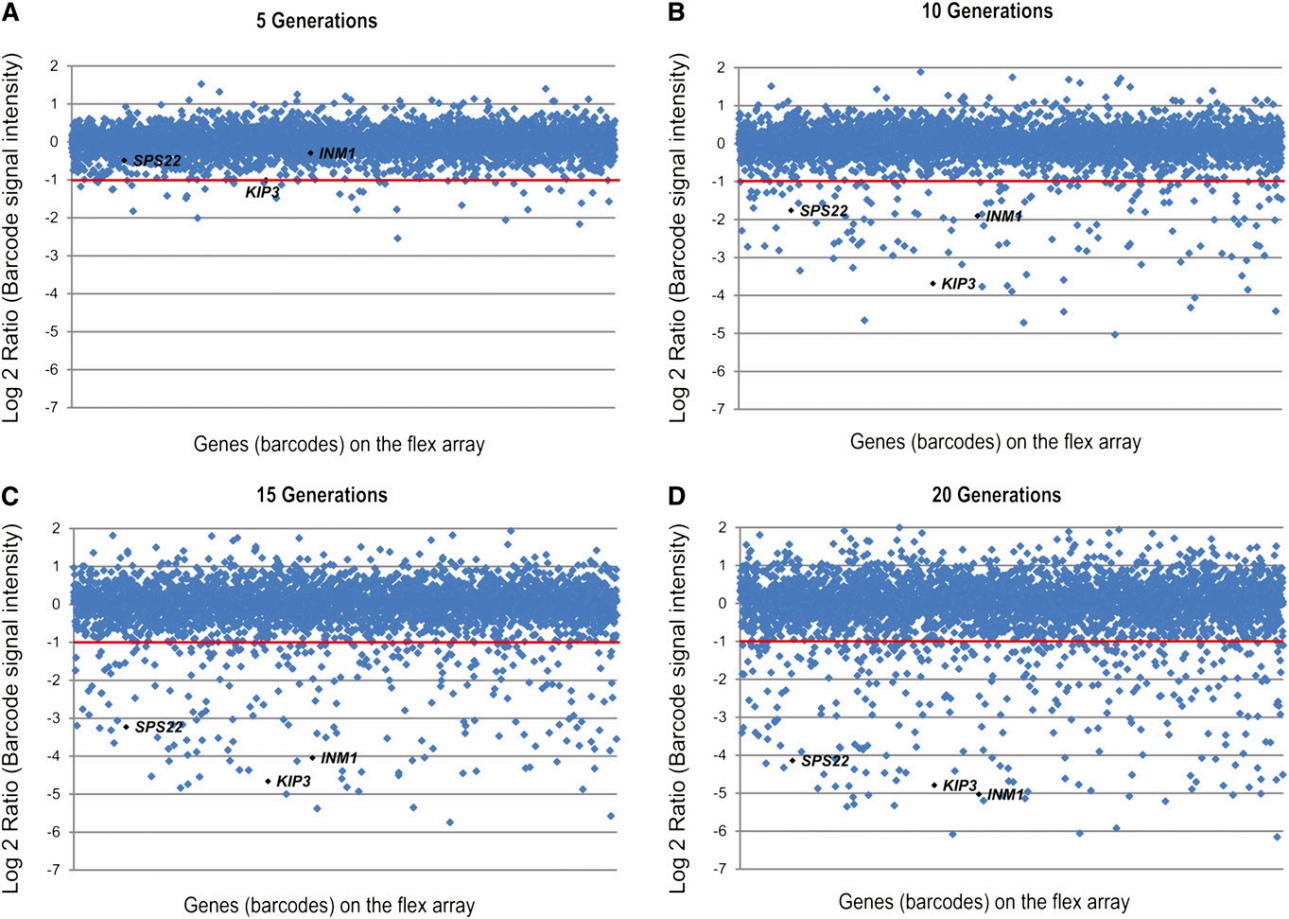
Comparison of toxicity in pooled cultures after 5 (A), 10 (B), 15 (C), and 20 (D) generations of growth. Scatterplots depict all barcode Log_2_ ratios of signal intensities that correspond to the fitness of each strain on the barFLEX array. Strains containing plasmids bearing toxic genes have a lower negative Log_2_ ratio. The greatest number of toxic genes are detected at 20 generations of growth. Genes are ordered on the X-axis according to their position on the barFLEX array. Genes referred to in the text are highlighted on the plots. Red line shows the cut off at Log2 ratio of −1.

Several groups have surveyed the yeast genome for genes that cause an obvious fitness defect when overexpressed (Gelperin *et al.* 2005; Sopko *et al.* 2006). All previous tests of overexpression toxicity have been performed by assaying growth on solid medium. We used our barFLEX collection to explore overexpression toxicity by manipulating pool aliquots in liquid medium as described previously (Ericson *et al.* 2010). We grew samples of the barFLEX pool in medium containing glucose (repressing) or galactose (inducing) and then collected samples every five generations for measurement of relative strain abundance. After five generations of growth in galactose-containing medium, very few strains decreased in abundance in the pool (Figure 2A). However, over the next 10 to 15 generations of growth, a number of strains, including those carrying deletion alleles of *SPS22*, *KIP3*, and *INM1*, decreased in abundance and the changes were identified clearly by microarray analysis (Figure 2, B and C). At 20 generations of growth (which is roughly equivalent to a medium-sized individual colony on an agar plate), more than 360 genes exhibited decreased abundance within the pool (Figure 2D; Table S10). Replicates of this experiment correlated well (r = 0.7897) and the variance further decreased when only the highly toxic genes (defined as those with Log_2_ ratio < −1) were considered (r = 0.847).

**Figure 3:**
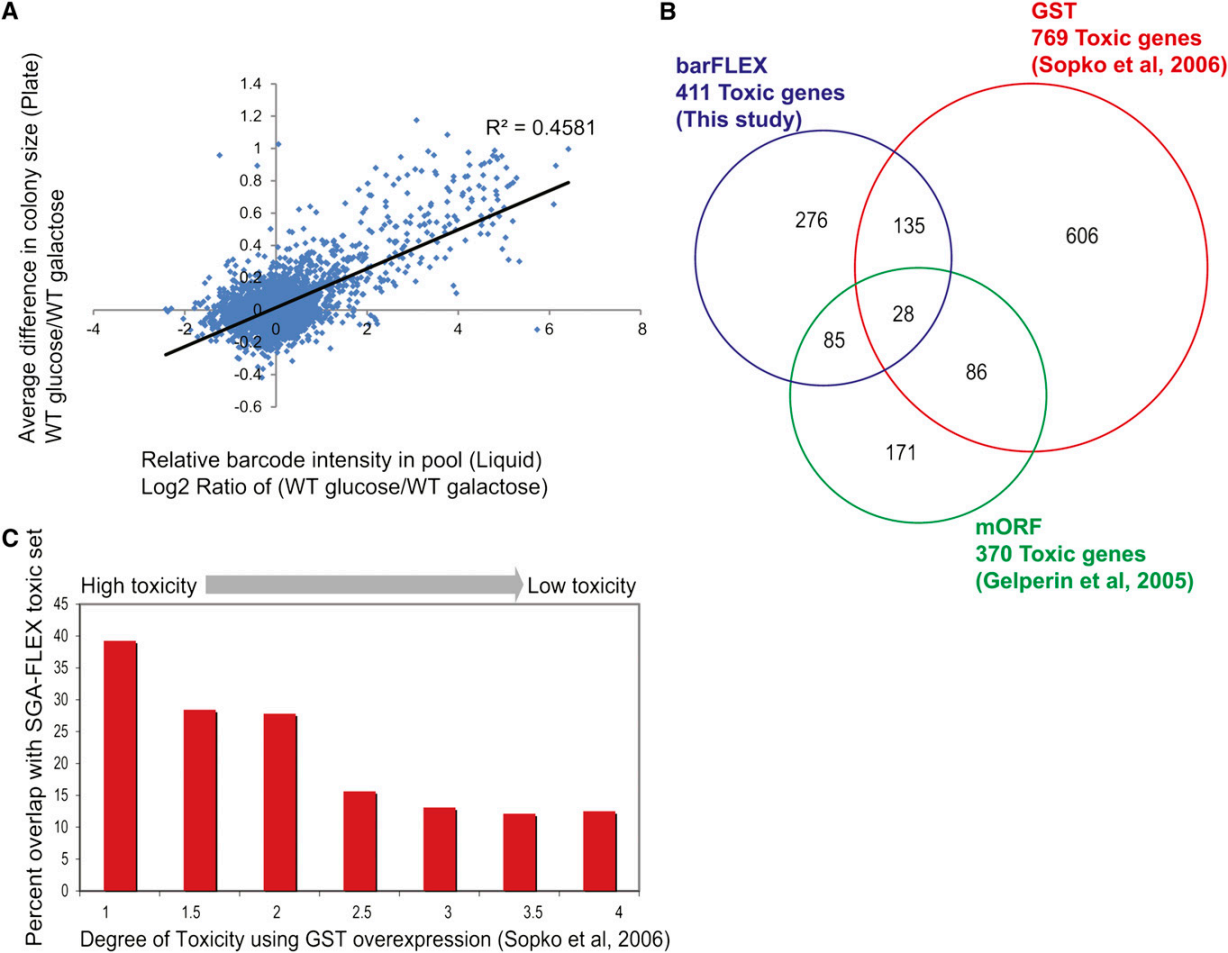
Characterization of the barFLEX toxic genes. (A) Comparison of barFLEX toxic lists collected using pooled growth *vs.* growth in solid medium. Log_2_ ratio of barcode intensities are plotted against colony fitness scores. (B) Venn Diagram of overlap between toxic lists in barFLEX, GST, and mORF collections. Three different genomic yeast collections were tested for genes that cause slow growth phenotypes when overexpressed. The FLEX and GST collections were tested on solid growth media + galactose to induce overexpression. The mORF collection was tested on solid media + galactose + glycerol + ethanol. (C) Overlap of GST and barFLEX by degree of toxicity. The overlap between only the toxic genes in the GST-ORF and barFLEX collection is shown. The GST-ORF collection was scored for toxic genes by serial spot dilution. A scale from 1 to 4 was used to qualitatively describe the fitness defect caused by overexpressing each gene (Sopko *et al.* 2006).

We compared the growth of the barFLEX collection in pooled liquid culture to growth of the array on plates. We examined the collection on solid medium for defects in growth by measuring colony size differences on glucose- or galactose-containing medium. The barFLEX array was pinned in a 1536 strain format onto glucose- and galactose-containing plates and colony size was assessed using an automated colony scoring method (Tong *et al.* 2004). This analysis was undertaken to: (1) assess the quality of the array, that is, whether barcodes and plasmids were assembled correctly; (2) validate the array for use in a pooled culture format and; (3) compare our results with previous measurements of gene toxicity on solid medium. In total, 411 genes in the barFLEX array appeared to cause a slow-growth phenotype using colony size as a proxy for fitness (Table S11). Despite the obvious technical differences between the two experiments, the competitive growth assay of overexpression toxicity correlated significantly with the measurements of toxicity using growth on solid medium (R = 0.68, R^2^ = 0.46; Figure 3A). Only 6 of the 65 genes that were considered toxic on solid medium did not meet the cutoff that we used to identify a toxic gene in our pooled liquid growth assay (Log_2_ ratio < −1). Also, only 4 of the 95 genes that caused a significant growth defect when overexpressed in liquid medium (Log_2_ < −3) were not obviously toxic in our plate-based assay. Genes that were highly toxic upon overexpression as assessed in our competitive liquid growth assay (Log_2_ < −4, representing a 16-fold difference in abundance) were also identified in the plate assay, with a false-positive rate of 2% (Table S12). As expected, the false-positive rate increased as the toxicity decreased, presumably due to the increased experimental variability associated with assessing a more subtle phenotype.

### Comparing barFLEX toxic set to other datasets of toxic genes

As noted previously our analysis of the barFLEX collection revealed 411 genes that caused a sick or lethal phenotype when overexpressed, using colony-size as a measure for fitness (Table S11). Previous assessment of the GST-ORF collection (Sopko *et al.* 2006) revealed a toxic set of 769 genes, and another study of the mORF GAL-ORF overexpression collection (Gelperin *et al.* 2005) identified 370 toxic genes. Comparing these results, we found that the toxic genes sets associated with any two collections overlaps by ~25% (Figure 3B), differences that presumably reflect the unique features of each collection and the unique assays used for assessing toxicity. Key array features to consider include: (1) the low-copy centromeric plasmid backbone (CEN) of the FLEX collection *vs*. the high-copy backbone (2 μ) of the GST collection; (2) the quality of each ORF in each collection (sequenced verified in the FLEX collection but not in others); (3) the particular strain background used and; (4) differences in the assays used to measure gene toxicity. For example, the mORF collection was scored for overexpression toxicity by analyzing the size of the colony resulting from spotting a single 3μL culture onto solid medium (Gelperin *et al.* 2005).

In contrast, the GST-ORF toxic set was identified by serial spot dilution assays and genes were assigned a qualitative score ranging from 1 (lethal) to 4 (least sick) (Sopko *et al.* 2006) while we measured growth of the barFLEX collection by pinning the array and measuring colony size using an automated scoring method. The degree of overlap between toxic gene lists correlated with the degree of toxicity as determined by serial spot dilution of the GST collection, with close to 40% overlap in the most toxic genes, down to ∼10% overlap in the less toxic genes (Figure 3C). This result is expected if the most toxic genes (Table S10 and Table S12) are more easily detected using different assays (Table S13).

Previous analysis of genes that cause fitness defects when overexpressed revealed a significant functional enrichment for genes with annotated roles in cell cycle regulation and mitosis (Gelperin *et al.* 2005; Sopko *et al.* 2006). The barFLEX toxic gene set was similarly enriched for cell cycle genes [*e.g.*, mitosis (*P* = 1.232 × 10^−8^) and genes involved in cell cycle regulation [*P* = 2.986 × 10^−8^ (Robinson *et al.* 2002)]. Genes that were uniquely toxic in the GST-ORF collection were also enriched for genes with roles in intracellular protein transport (*P* = 6.91 × 10^−5^), which we did not observe with the barFLEX collection, suggesting the GST-ORF enrichment may reflect an effect of the GST tag on protein transport that is less likely to be seen with untagged genes expressed in the barFLEX collection.

### Using the barFLEX collection for SDL screening in a pooled format

As noted previously, overexpression of most genes in WT cells is phenotypically benign, but sensitivity to gene dosage can be revealed in strains mutated for an interacting protein or pathway component, a so-called SDL interaction. To develop protocols for using the barFLEX collection for identifying SDL interactions, we performed an SDL screen using a query strain deleted for the *DUN1* kinase. We chose the *dun1Δ* strain for our test screens since it is conditionally activated by DNA damage and has been previously screened using colony size measurements on solid medium and the GST-tagged overexpression array (Sharifpoor *et al.* 2012). Dun1 responds to DNA damage downstream of the Rad53 and Mec1 kinases and phosphorylates Rad55, a protein involved in double strand break repair (Bashkirov *et al.* 2003; Zhao and Rothstein 2002). We generated a *dun1Δ* query strain marked with a *natMX* antibiotic resistance cassette in a *MAT***a** BY4741 background (lacking SGA mating type selectable markers). We then used SGA to introduce the kinase deletion allele into the barFLEX collection and selected for haploids carrying the overexpression plasmid, the integrated barcode, and the deletion allele (Figure S3). After the final selection, we pooled the strains and cultured them alongside a negative control strain (*ura3Δ*::*natMX*) in glucose- or galactose-containing medium for a total of 20 generations (Figure S3).

**Figure 4:**
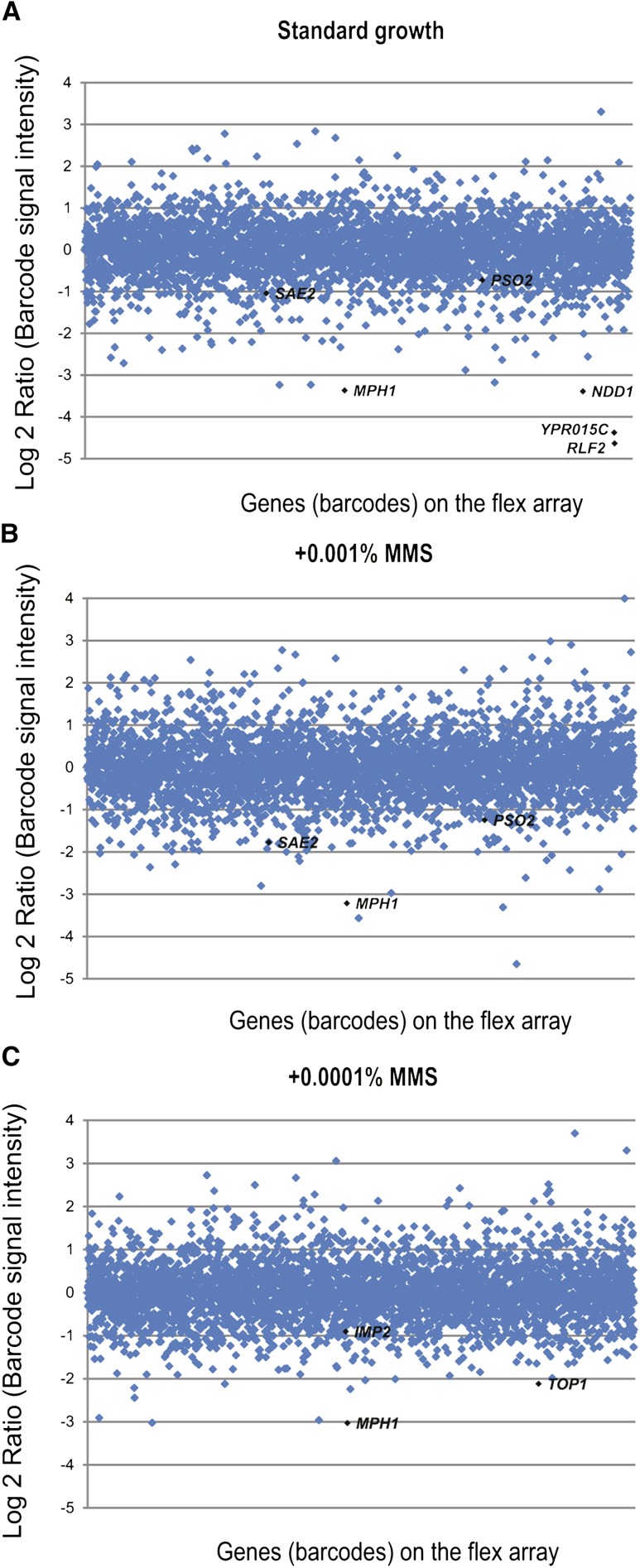
Assessment of SDL interactions in a *dun1* deletion using the barFLEX collection in pooled cultures under standard growth conditions or in the presence of 0.001% and 0.0001% MMS. Scatterplots depict all SDL interactions identified using *dun1*Δ in standard growth conditions (A) or in the presence of 0.001% MMS (B) and 0.0001% MMS (C). Log_2_ ratios of barcode signal intensities corresponding to fitness of each strain when overexpressed in the *dun1*Δ are plotted. Genes are ordered in the X-axis according to their position on the barFLEX array. Genes referred to in the text are highlighted on the plots.

**Figure 5:**
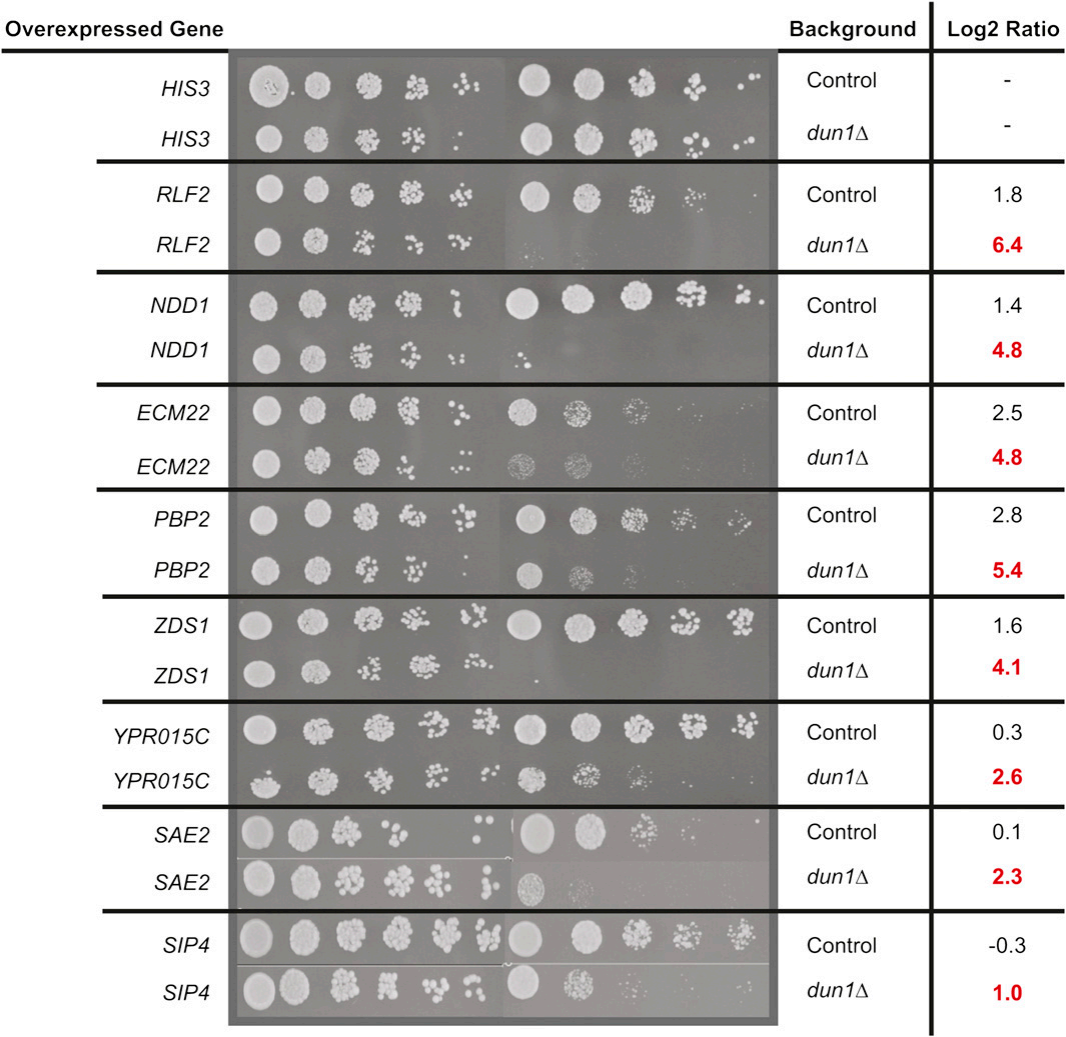
Confirmed SDL interactions for *dun1* using serial spot dilutions. Eight confirmed SDL interactions with *DUN1* are shown. Cells were diluted 15-fold at each step and spotted on either glucose (left) or galactose (right) to induce overexpression. Corresponding Log_2_ ratios of barcode intensities in both control (black) and *dun1*Δ (red) strains are shown.

To identify SDL interactions, we compared the pooled growth ratio of each barcoded strain that also carried our deletion of interest in glucose/galactose after 20 generations (cutoff Log_2_ ratio < −1), eliminating any genes that were also toxic in the negative control screen (difference of Log_2_ ratio < −1). Using this statistical cut-off, the *dun1Δ* screen revealed a list of 52 interactions (Figure 4A; Table S14), 25 of which have at least one phosphorylated residue (Amoutzias *et al.* 2012). The SDL interactions included genes encoding two known downstream proteins *RLF2*, which encodes a member of the CAF-1 chromatin assembly complex, and *NDD1*, which encodes a transcriptional activator of S-phase genes, as the top hits. We also identified genes known to be involved in DNA repair, such as *MPH1*, a 3′-5′ DNA helicase involved in error-free bypass of DNA lesions, and previously uncharacterized genes, *e.g.*, *YPR015C*. We confirmed all of the SDL interactions identified from the *dun1*Δ pooled screens using either serial spot dilution as previously described (Sharifpoor *et al.* 2012) or using automated liquid growth curve assays (see *Materials and Methods*). We used stringent criteria for identifying an SDL interaction in our secondary assays; we demanded an obvious growth difference that was clear over two 10-fold serial spot dilutions or a >10% difference in growth fitness scores, with the fitness defect larger than twice the standard deviation between the control and the kinase deletion strain (St Onge *et al.* 2007). Using these assays, we confirmed 20 of the interactions for *dun1*Δ using either spot dilutions or automated liquid growth curves (Figure 5; Table S14), corresponding to a confirmation rate of ~38%. This confirmation rate is less than that seen in large-scale SDL screens of the kinome [46% (Sharifpoor *et al.* 2012)] using a colony-based readout and likely reflects a combination of factors, including our stringent cut-off, which affects sensitivity, and innate differences in the assay conditions.

Screening the kinome using traditional SDL screening approaches revealed that many kinase deletion alleles show few or no SDL interactions (Sharifpoor *et al.* 2012). To test the utility of the barFLEX array for revealing condition-specific SDL interactions, we next treated the *dun1*Δ-barFLEX pool with a sublethal dose of the DNA methylating agent MMS (Figure 4, B and C). We examined both a control (*ura3*Δ-barFLEX*)* and the *dun1*Δ-barFLEX collections throughout 20 generations of growth in three different conditions: (1) galactose (to induce overexpression) for 20 generations; (2) galactose for five generations of growth followed by addition of 0.0001% MMS for 15 generations; (3) galactose for five generations followed by addition of 0.001% MMS for five generations to a total of 20 generations. Glucose (noninduced) controls were performed in parallel. The dose of MMS used in the experiment was selected to induce only a slight fitness defect in the *dun1Δ* strain. In the presence of 0.001% MMS, the WT (*ura3Δ*) screen identified 197 additional toxic genes (Log_2_ < −1; Figure 4B; Table S15) including known repair genes *SAE2*, *MPH1*, and *PSO2*, which is consistent with published chemogenomic data (Lee *et al.* 2005). At 0.0001% MMS, 135 additional toxic genes were identified (Figure 4C; Table S16), including *MPH1*, as well as other DNA repair genes such as *TOP1* and *IMP2* (Downs *et al.* 2000; Masson and Ramotar 1996). The *dun1Δ* screen in the presence of either concentration of MMS identified a number of condition-specific SDL interactions (77 additional genes for 0.001% MMS and 45 additional genes for 0.0001% MMS), including interactions with the known checkpoint gene *LCD1* and the repair gene *RAD26* (Table S17 and Table S18). The strain overexpressing the known target of Dun1, Sml1, decreased in strain abundance in all three of the conditions compared with WT but was below the stringent statistical cut-off that we chose for our experiments.

**Figure 6:**
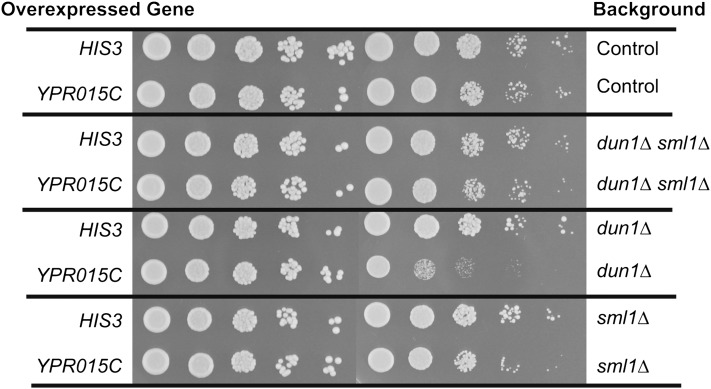
Deletion of *SML1* suppresses the toxicity caused by the overexpression of *YPR015C* in a *dun1*Δ background. Serial 15-fold dilutions of each strain were spotted on (right) glucose- and (left) galactose-containing plates to induce overexpression.

We were intrigued by our discovery of a previously uncharacterized gene, *YPR015C*, which encodes a zinc finger transcription factor, as an SDL interaction with *DUN1* deletion in both standard growth and in DNA damage-inducing conditions. In WT cells, Dun1 regulates the levels of ribonucleotide reductase gene expression (*RNR2*, *RNR3*, and *RNR4*) in both standard growth conditions and in the presence of DNA damage through degradation of the ribonucleotide reductase inhibitor Sml1 (Elledge *et al.* 1993; Zhao and Rothstein 2002; Zhou and Elledge 1993). Dun1 also acts with Mec1 and Rad53 to phosphorylate a repressor of *RNR* transcription, Crt1 (Huang *et al.* 1998). Phosphorylation of Crt1 leads to derepression of *RNR* gene expression as well as expression of *YPR015C*. Overexpression of *YPR015C* causes up-regulation of *RNR* genes (Chua *et al.* 2006), suggesting that *YPR015C* may be involved in the DNA damage checkpoint. Finally, *YPR015C* overexpression causes a cell cycle delay in G2 (Niu *et al.* 2008), as well as a mildly toxic phenotype in the *SML1* deletion strain (Figure 6). To ask whether *YPR015C* acts in the same pathway as Dun1-Sml1, we constructed an *sml1*Δ*dun1*Δ double-mutant strain carrying a plasmid expressing *YPR015C* from the inducible GAL promoter. Consistent with the known suppression of *DUN1* mutant phenotypes by deletion of *SML1*, we saw that deletion of *SML1* also partially suppressed the SDL phenotype caused by overexpression of *YPR015C* in the *dun1*Δ mutant (Figure 6). This result is consistent with our finding that *YPR015C* is less toxic when overexpressed in *sml1*Δ, compared with the *dun1*Δ background (Figure 6). Together, these results suggest that the toxicity caused by the overexpression of *YPR015C* in the *dun1Δ* is likely due to increased expression of the *RNR* genes.

To summarize, induction of gene overexpression using nutrient-inducible promoters (*e.g.*, galactose-inducible promoters) has enabled functional assessment of genetic perturbations for gain-of-function alleles (Chua *et al.* 2004; Gelperin *et al.* 2005; Sopko *et al.* 2006). A modification of this approach uses a chimeric transcriptional activator that fuses the DNA-binding domain of Gal4 to the estrogen receptor and the VP16 activator, allowing rapid and specific induction of individual genes using the hormone, β-estradiol (McIsaac *et al.* 2011). Such fast-acting and graded promoters enable dynamic assessment of overexpression phenotypes resulting from mis-regulation of complex and presumably highly buffered regulatory networks. Here, we describe development of the new barFLEX array that allows for assessment of overexpression phenotypes in pooled liquid cultures. We validated the barFLEX collection for facile detection of genes exhibiting conditional toxicity and for SDL screening in liquid cultures. Although simple in design, testing WT backgrounds in different conditions promises to reveal new gene functions. In addition, the full Barcoder reagent set we describe is amenable to diverse applications and can be used to barcode other plasmids or mutant collections for high throughput experiments, including those involving other overexpression systems (McIsaac *et al.* 2011).

In addition to screening for fitness defects caused by gene overexpression in WT cells, we also validated the barFLEX collection for detection of genes exhibiting conditional SDL using kinases as a test case. By perturbing a barFLEX pool carrying a deletion of the kinase gene *DUN1* with the DNA damaging agent MMS, we uncovered novel genetic interactions that were not apparent in standard conditions. This general approach will be extremely valuable for studying kinases that are known to be conditionally active and other regulators that are required under particular conditions. Conditions that might be useful for SDL and other overexpression screens could be identified by surveying data from systematic phenotypic analysis of the deletion collection (Hillenmeyer *et al.* 2008) or from large genetic interaction datasets that may indicate a genetic background suitable for screening (Costanzo *et al.* 2010).

## Supplementary Material

Supporting Information
