# Supplementary material for: Functional Analysis With a Barcoder Yeast Gene Overexpression System
Source: G3 (Bethesda). 2012 Oct 1;2(10):1279–89. doi: 10.1534/g3.112.003400 (PMC3464120; doi:10.1534/g3.112.003400)
Supplement: Supporting Information [file supp_2_10_1279__index.html]

Supporting Information 

# Functional Analysis With a Barcoder Yeast Gene Overexpression System

## Supporting Information for Douglas *et al.*, 2012

**Files in this Data Supplement:**

- Supporting Information - Figures S1-S3 and Tables S1-S18 (PDF, 114 KB)
- Figure S1 - Theory of Synthetic Dosage Lethality (PDF, 64 KB)
- Figure S2 - Universal primers (A) and strategy (B) used for the construction of barcoder strains (PDF, 57 KB)
- Figure S3 - Methodology for conditional SDL screening in pooled culture (PDF, 78 KB)
- Table S1 - List of Yeast Strains used in this study (.xlsx, 10 KB)
- Table S2 - List of ORFs represented on the SGA-FLEX array (.xlsx, 152 KB)
- Table S3 - List of ORFs represented on the Barcoder array (.xlsx, 662 KB)
- Table S4 - List of strains tested by PCR confirmation and sequencing of both the ORFs and barcodes (.xlsx, 42 KB)
- Table S5 - List of ORFs represented on the barFLEX array (.xlsx, 791 KB)
- Table S6 - Percentage of all barFLEX strains with detectable barcode tags assessed by hybridization data (.xlsx, 51 KB)
- Table S7 - List of undetected barcodes (.xlsx, 165 KB)
- Table S8 - Percentage of all barFlex strains with identifiable barcode tags, assessed by NextGen Sequencing (.xlsx, 25 KB)
- Table S9 - Identification of Barcode Sequences by NextGen Sequencing (.xlsx, 531 KB)
- Table S10 - List of barcode readouts for all genes on the barFLEX with the TOXIC genes highlighted (Log2 ratio) (.xlsx, 203 KB)
- Table S11 - List of toxic genes on the barFLEX using colony size measurements as a proxy for fitness (.xlsx, 79 KB)
- Table S12 - False Positive Rates for Identifying Toxic Genes in Liquid Growth Medium by comparison to plate-based assay (.xlsx, 51 KB)
- Table S13 - Overlapping toxic genes with GST collection (.xlsx, 49 KB)
- Table S14 - Dun1 SDL hits in standard growth conditions (.xlsx, 59 KB)
- Table S15 - Wild type toxic list in the presence of 0.001% MMS (5 generation of treatment) (.xlsx, 75 KB)
- Table S16 - Wild type toxic list in the presence of 0.0001% MMS (15 generation of treatment) (.xlsx, 48 KB)
- Table S17 - Dun1 SDL hits in the presence of 0.001% MMS (5 generations of treatment) (.xlsx, 61 KB)
- Table S18 - Dun1 SDL hits in the presence of 0.0001% MMS (15 generation of treatment) (.xlsx, 62 KB)
