# Supplementary material for: Functional Analysis With a Barcoder Yeast Gene Overexpression System
Source: G3 (Bethesda). 2012 Oct 1;2(10):1279–89. doi: 10.1534/g3.112.003400 (PMC3464120; doi:10.1534/g3.112.003400)
Supplement: Supporting Information [file supp_2.10.1279_003400SI.pdf]

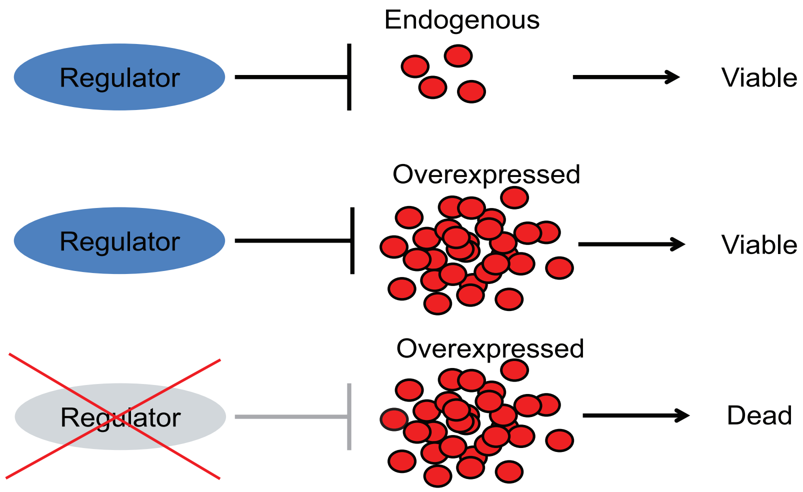


**Figure S1** Theory of Synthetic Dosage Lethality. A conceptual framework for interpreting synthetic dosage lethal (SDL) interactions is depicted. Proteins that regulate downstream signaling through other proteins do not always cause changes in cell viability when deleted. Similarly, when the downstream protein is overexpressed, cell viability may be unaffected. A synthetic dosage lethal interaction may occur when the combination of these two perturbations creates a non-viable or sick cell due to a misregulated downstream protein (A). For example if a negative regulator is deleted and an opposing activating protein is overexpressed, lethality may occur (B). SDL interactions have identified direct substrates of kinases (A) but may also identify proteins in opposing pathways or proteins regulated by a direct substrate of the regulator.

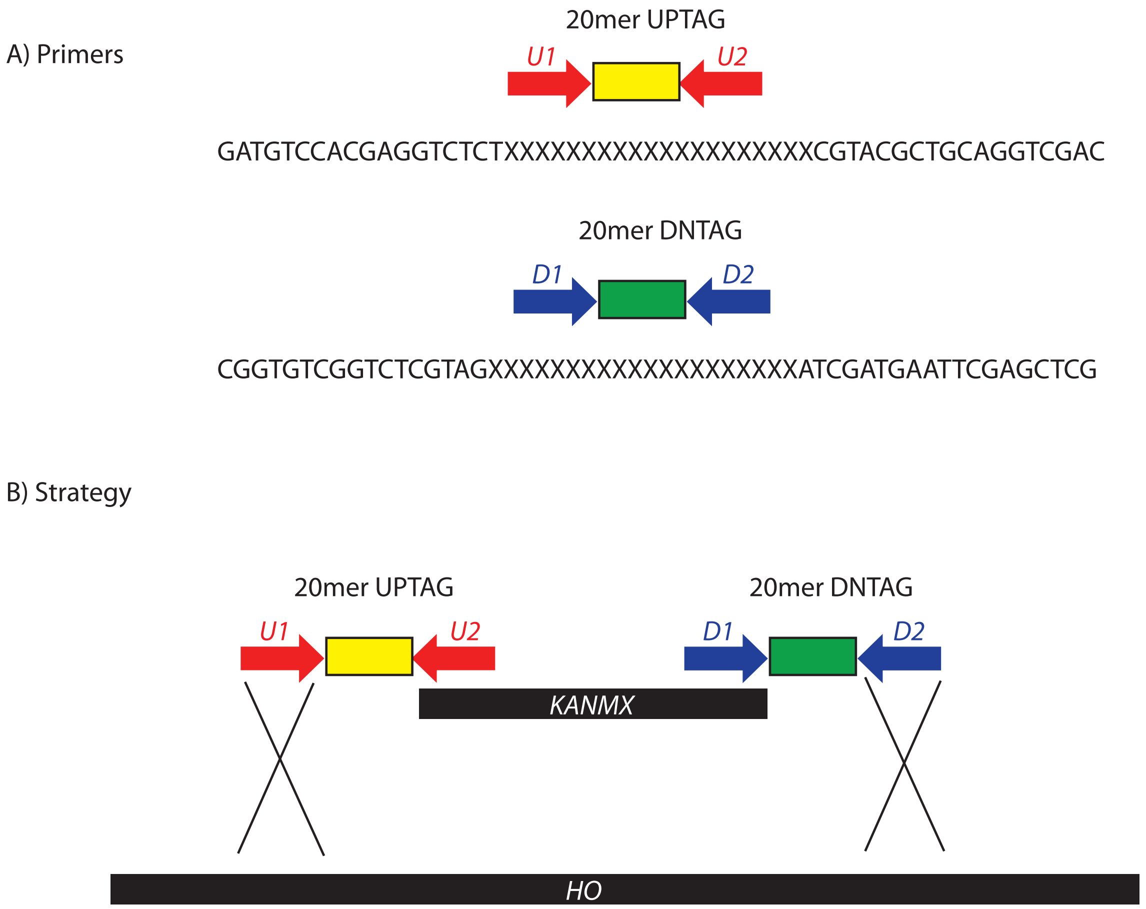


**Figure S2** Universal primers (A) and strategy (B) used for the construction of barcoder strains. UPTAG and DOWNTAG primer pairs used to amplify the kanamycin resistance cassette with 20-mer barcodes are shown. Primers U2 and D1 are homologous to the *kanMX* cassette, and U1 and D2 are homologous to the *ho* locus. PCR reactions were performed using Platinum PCR Supermix High Fidelity (Invitrogen). The transformants were selected by replica-plating onto standard yeast peptone dextrose (YPD) + G418.

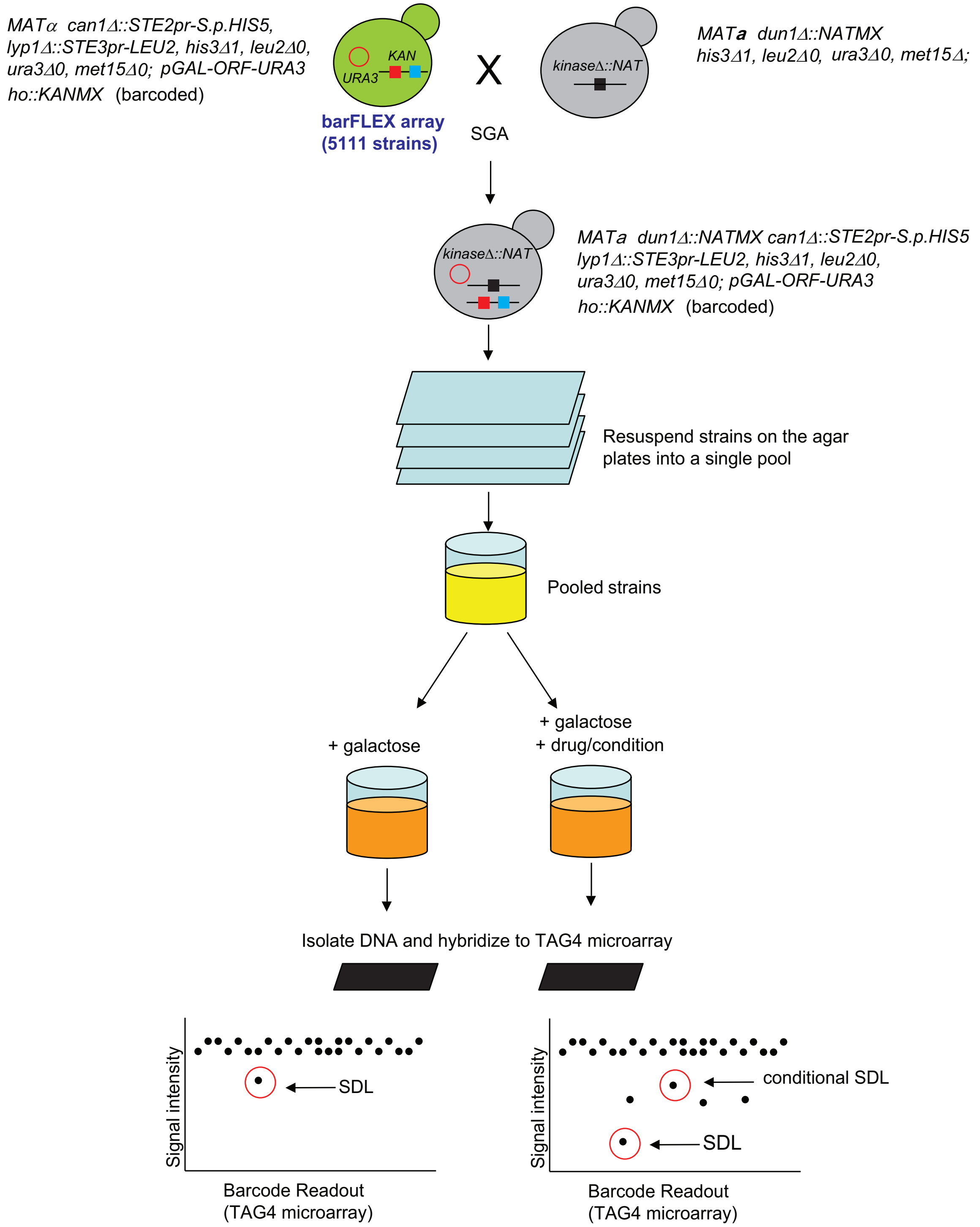


**Figure S3** Methodology for conditional SDL screening in pooled culture. Kinase deletion alleles marked with *natMX* can be introduced into the barFLEX collection through SGA. Standard methods for competitive pooled growth experiments are then used to test the novel strains for SDL interactions. The pooled strains can be subjected to a variety of stressors. This methodology ensures that every plasmid is present in the initial pool of strains. Induction of overexpression in galactose-containing media then allows for identification of SDL interactions. PCR amplification of the barcodes in the pool allows for quantification of the relative presence of a particular strain in the pool. The overall growth of each strain is measured by a barcode read-out on a TAG4 microarray.

**Supporting Tables**

Available for download at http://www.g3journal.org/lookup/suppl/doi:10.1534/g3.112.003400/-/DC1.

Table S1- List of Yeast Strains used in this study
Table S2- List of ORFs represented on the SGA-FLEX array
Table S3- List of ORFs represented on the Barcoder array
Table S4- List of strains tested by PCR confirmation and sequencing of both the ORFs and barcodes
Table S5- List of ORFs represented on the barFLEX array
Table S6- Percentage of all barFLEX strains with detectable barcode tags assessed by hybridization data
Table S7- List of undetected barcodes
Table S8- Percentage of all barFlex strains with identifiable barcode tags, assessed by NextGen Sequencing.
Table S9- Identification of Barcode Sequences by NextGen Sequencing
Table S10- List of barcode readouts for all genes on the barFLEX with the TOXIC genes highlighted ($Log_2$ ratio)
Table S11- List of toxic genes on the barFLEX using colony size measurements as a proxy for fitness
Table S12- False Positive Rates for Identifying Toxic Genes in Liquid Growth Medium by comparison to plate-based assay
Table S13- Overlapping toxic genes with GST collection
Table S14: Dun1 SDL hits in standard growth conditions
Table S15: Wild type toxic list in the presence of 0.001% MMS (5 generation of treatment)
Table S16: Wild type toxic list in the presence of 0.0001% MMS (15 generation of treatment)
Table S17: Dun1 SDL hits in the presence of 0.001% MMS (5 generations of treatment)
Table S18: Dun1 SDL hits in the presence of 0.0001% MMS (15 generation of treatment)

WEBLINKS
All supplementary Figures, Tables and the list of sequences for all reported barcodes can be accessed at the following link:

http://chemogenomics.med.utoronto.ca/supplemental/barflex/
