# Supplementary material for: Functional Analysis With a Barcoder Yeast Gene Overexpression System
Source: G3 (Bethesda). 2012 Oct 1;2(10):1279–89. doi: 10.1534/g3.112.003400 (PMC3464120; doi:10.1534/g3.112.003400)
Supplement: Supporting Information [file supp_2.10.1279_FigureS1.pdf]

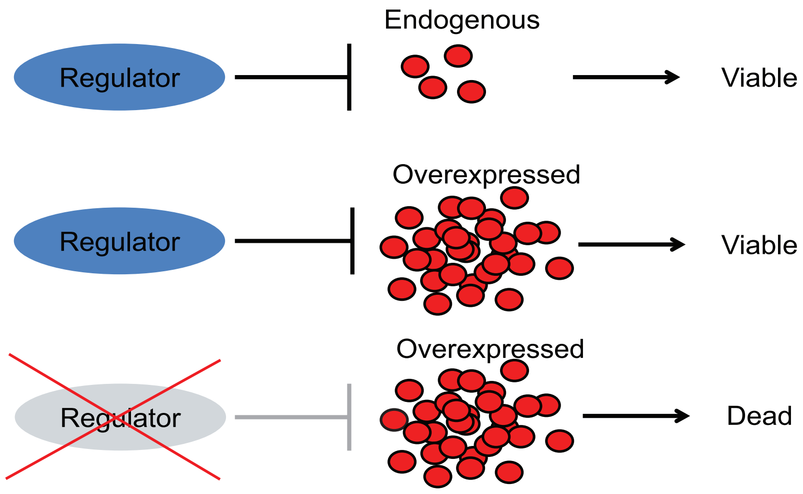

Figure S1 Theory of Synthetic Dosage Lethality. A conceptual framework for interpreting synthetic dosage lethal (SDL) interactions is depicted. Proteins that regulate downstream signaling through other proteins do not always cause changes in cell viability when deleted. Similarly, when the downstream protein is overexpressed, cell viability may be unaffected. A synthetic dosage lethal interaction may occur when the combination of these two perturbations creates a non-viable or sick cell due to a misregulated downstream protein (A). For example if a negative regulator is deleted and an opposing activating protein is overexpressed, lethality may occur (B). SDL interactions have identified direct substrates of kinases (A) but may also identify proteins in opposing pathways or proteins regulated by a direct substrate of the regulator.
