# Supplementary material for: Functional Analysis With a Barcoder Yeast Gene Overexpression System
Source: G3 (Bethesda). 2012 Oct 1;2(10):1279–89. doi: 10.1534/g3.112.003400 (PMC3464120; doi:10.1534/g3.112.003400)
Supplement: Supporting Information [file supp_2.10.1279_FigureS2.pdf]

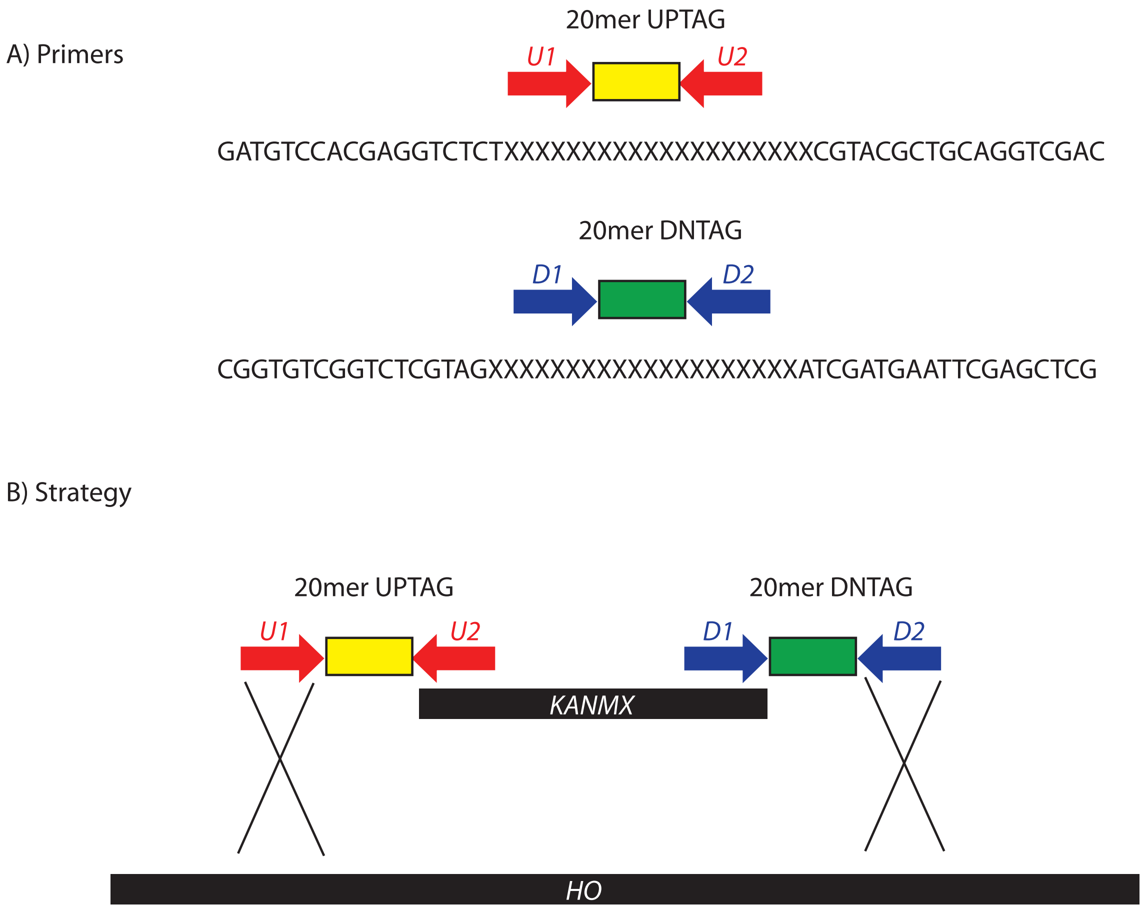

Figure S2 Universal primers (A) and strategy (B) used for the construction of barcoder strains. UPTAG and DOWNTAG primer pairs used to amplify the kanamycin resistance cassette with 20-mer barcodes are shown. Primers U2 and D1 are homologous to the *kanMX* cassette, and U1 and D2 are homologous to the *ho* locus. PCR reactions were performed using Platinum PCR Supermix High Fidelity (Invitrogen). The transformants were selected by replica-plating onto standard yeast peptone dextrose (YPD) + G418.
