# Supplementary material for: Functional Analysis With a Barcoder Yeast Gene Overexpression System
Source: G3 (Bethesda). 2012 Oct 1;2(10):1279–89. doi: 10.1534/g3.112.003400 (PMC3464120; doi:10.1534/g3.112.003400)
Supplement: Supporting Information [file supp_2.10.1279_FigureS3.pdf]

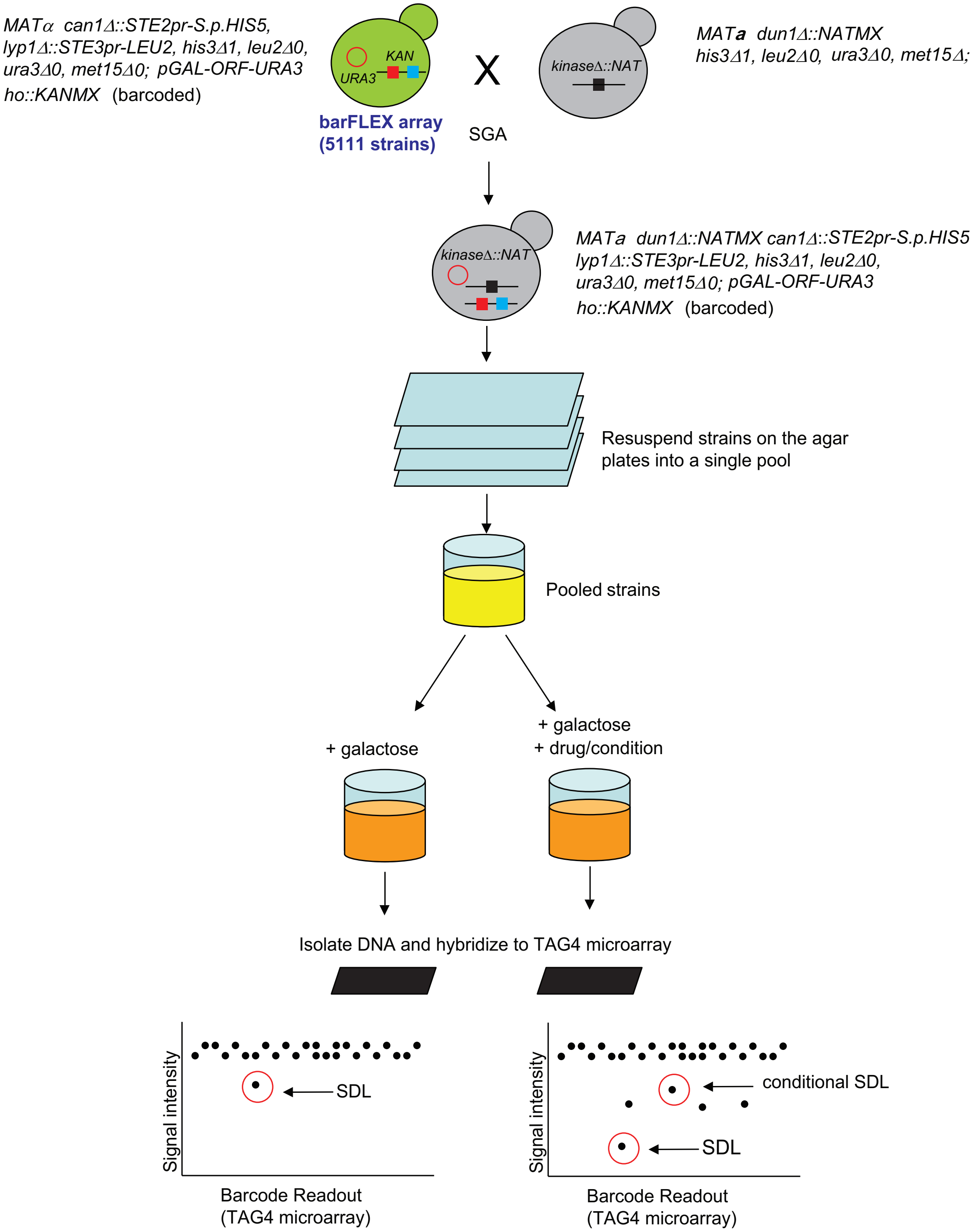

Figure S3 Methodology for conditional SDL screening in pooled culture. Kinase deletion alleles marked with *natMX* can be introduced into the barFLEX collection through SGA. Standard methods for competitive pooled growth experiments are then used to test the novel strains for SDL interactions. The pooled strains can be subjected to a variety of stressors. This methodology ensures that every plasmid is present in the initial pool of strains. Induction of overexpression in galactose-containing media then allows for identification of SDL interactions. PCR amplification of the barcodes in the pool allows for quantification of the relative presence of a particular strain in the pool. The overall growth of each strain is measured by a barcode read-out on a TAG4 microarray.
